# Reviewed article: Freitas TCR, Soares AB, Figueiredo BNS. Evaluation of prophylaxis protocols and spatial distribution of humans wounded by bats: a descriptive study, Palmas, 2013-2023. Epidemiol Serv Saude. 2026:35;e20250557

**DOI:** 10.1590/S2237-96222026v35e20250557.a

**Published:** 2026-03-16

**Authors:** Kellyn Kessiene de Sousa Cavalcante

**Affiliations:** 1 Universidade Federal do Ceará, Fortaleza, CE, Brazil

## Peer review A

## Questionnaire

Does the manuscript contain new and significant information to justify publication? Yes

Does the Abstract (Summary) clearly and accurately describe the content of the article? Yes

Is the problem significant and concisely stated? Yes

Are the methods described comprehensively? No

Are the interpretations and conclusions justified by the results? No

Is adequate reference made to other work in the field? Yes

## Manuscript Structure

Length of article is: Too short

Number of tables is: Adequate

Number of figures is: Adequate

Please state any conflict(s) of interest that you have in relation to the review of this paper. None

Interest: Good

Quality: Average

Originality: Average

Overall: Average

Recommendation: Minor Revision

## Comments

Seguem sugestões para melhorar o artigo:

- Resumo: Incluir informações nos métodos (variáveis selecionadas e fonte de dados); - Introdução: focar mais no assunto do artigo;

- Métodos: Informar a versão do QGis para o georreferenciamento e do Tabwin para organização dos dados; inserir informações sobre os “aspectos éticos”.

- Resultados: Ajustar para “atendimentos antirrábicos” em vez de “casos de atendimentos antirrábicos”; inserir títulos de rótulos nos eixos x e y do gráfico; inserir a rosa dos ventos e a escala numérica nos mapas; organizar os dados em ordem decrescente na Tabela 1.

